# Timing and characteristics of nuclear events during conjugation and genomic exclusion in *Paramecium multimicronucleatum*

**DOI:** 10.1007/s42995-022-00137-y

**Published:** 2022-08-19

**Authors:** Xue Zhang, Xiaoteng Lu, Yong Chi, Yaohan Jiang, Chundi Wang, Saleh A. Al-Farraj, Adriana Vallesi, Feng Gao

**Affiliations:** 1 Institute of Evolution and Marine Biodiversity, Ocean University of China, Qingdao, 266003 China; 2 Key Laboratory of Mariculture (OUC), Ministry of Education, Qingdao, 266003 China; 3 Department of Biology, Shenzhen MSU-BIT University, Shenzhen, 518172 China; 4 Laboratory of Marine Protozoan Biodiversity and Evolution, Shandong University, Weihai, 264209 China; 5 Zoology Department, College of Science, King Saud University, Riyadh, 11451 Saudi Arabia; 6 Laboratory of Eukaryotic Microbiology and Animal Biology, University of Camerino, 62032 Camerino, Italy; 7 Laboratory for Marine Biology and Biotechnology, Pilot National Laboratory for Marine Science and Technology (Qingdao), Qingdao, 266237 China

**Keywords:** Amicronucleate cells, Nuclear development, Ciliate, Life cycle, Sexual reproduction

## Abstract

Ciliated protists are ideal material for studying the origin and evolution of sex, because of their nuclear dimorphism (containing both germline micronucleus and somatic macronucleus in the same cytoplasm), special sexual processes (conjugation and autogamy), and high diversity of mating-type systems. However, the study of sexual process is limited to only a few species, due to the difficulties in inducing or observing conjugation. In the present study, we investigate the conjugation process in *Paramecium multimicronucleatum*: (1) of the three prezygotic divisions, all micronuclei undergo the first two divisions (meiosis I, II), while a variable number of nuclei undergo the third division (mitosis); (2) the synkaryon divides three times after fertilization, giving rise to eight products that differentiate into four macronuclear anlagen and four micronuclei; (3) cells restore the vegetative stage after two successive cell fissions during which the macronuclear anlagen are distributed into daughter cells without division, while micronuclei divide mitotically; (4) the parental macronucleus begins to fragment following the first meiotic division and finally degenerates completely; (5) the entire process takes about 110 h, of which about 85 h are required for macronuclear development. In addition, we describe for the first time the process of genomic exclusion occurring between amicronucleate and micronucleate cells of *P. multimicronucleatum*, during which the micronucleate cell contributes a pronucleus to the amicronucleate cell, resulting in both exconjugants being homozygotes. These results provide new insights into the diversity of sexual processes and lay an important cytological basis for future in-depth studies of mating systems in ciliates.

## Introduction

The origin and maintenance of sex are regarded as “queen of problems in evolutionary biology” (Bell 1982): almost all eukaryotes, from uni- to pluricellular organisms engage in sex, a composite process including the formation of haploid gametes through meiosis, followed by the fusion of these gametes (fertilization). According to the eukaryotic fossil record, sex first appeared about two billion years ago in the single-celled common ancestor of eukaryotes (Zimmer 2009). In this context, ciliates represent excellent experimental models for studying the origin of sex: they are one of the most morphologically diverse and highly differentiated group among single-celled eukaryotic microorganisms and have been used as models for numerous studies in cell biology, genetics, genomics, and origin and evolution of eukaryotes (Cech 1985; Cheng et al. 2019; Greider and Blackburn 1985; Montagnes et al. 2012; Wang et al. 2021; Zhao et al. 2020). In addition, they share a special sexual process, known as conjugation (genetic recombination and nuclear reorganization occur without cell fusion), and a high diversity of mating-type (equivalent to gender) systems (Gao et al. 2020; Gong et al. 2020; Jiang et al. 2019; Orias et al. 2017; Phadke and Zufall 2009).

**Fig. 1:**
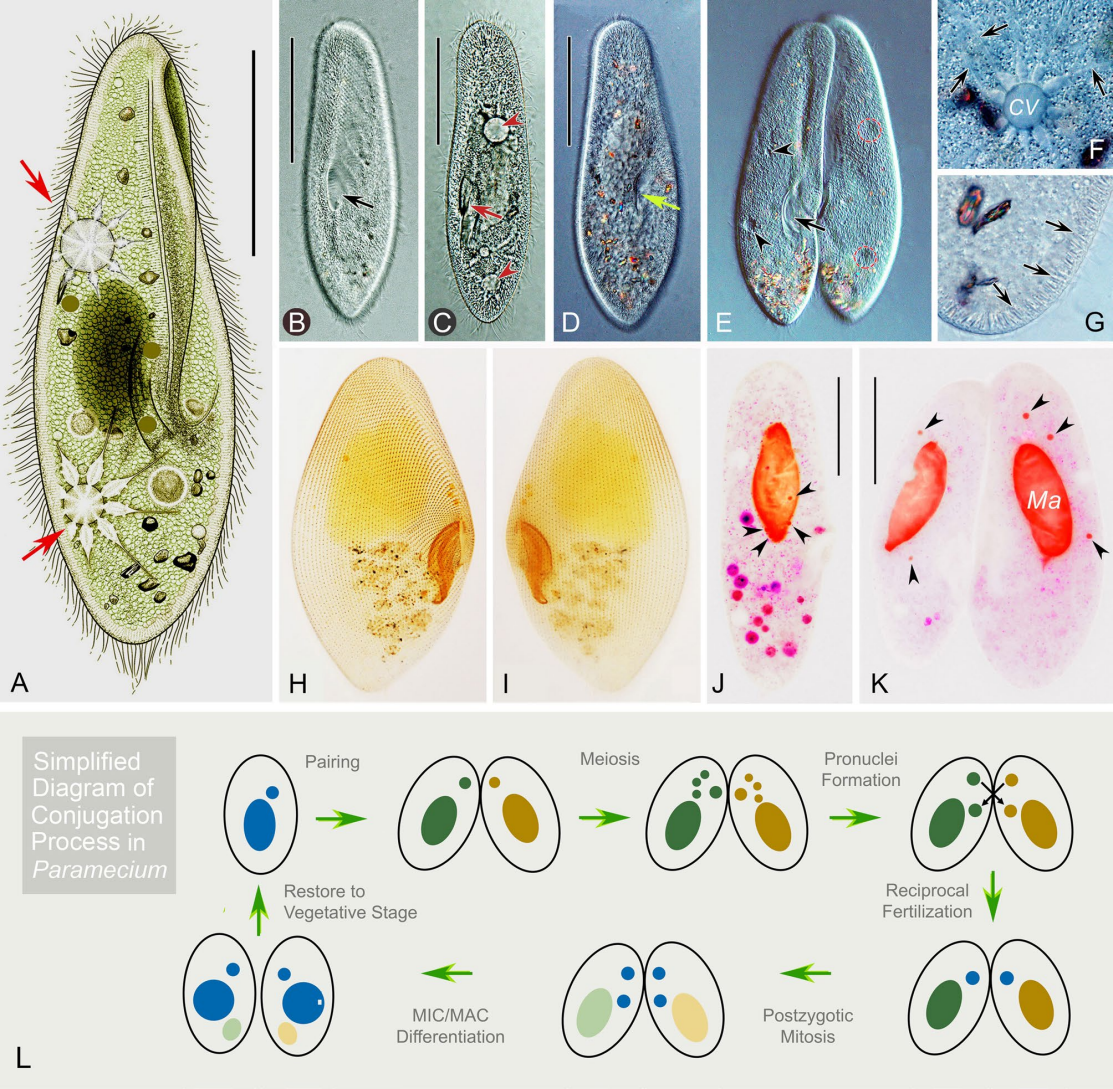
*Paramecium multimicronucleatum* in vivo (**A**–**G**), after silver carbonate impregnation (**H**, **I**), and after fluorescence staining by Hoechst 33342 and acridine orange (**J**, **K**). **A** Schematic diagram of ventral view of a typical cell; arrows mark the two contractile vacuoles. **B**–**E** Ventral views of different individuals (**B**–**D**) and a conjugated pair (**E**) observed in bright field (**C**) or differential interference contrast (DIC; **B**, **D**, **E**) showing the buccal field (arrows) and contractile vacuoles (arrowheads or red circle). **F** Details of a contractile vacuoles and collecting canals (arrows) observed by DIC. **G** Spindle-shaped extrusomes (arrows) beneath the pellicle observed by DIC. **H**, **I** Ventral and dorsal views of the same individual showing the ciliature after silver carbonate impregnation; arrow marks the buccal field. **J** A vegetative cell showing the macronucleus and micronuclei (arrowheads) after fluorescence staining by Hoechst 33342 and acridine orange. **K** A conjugated pair showing the macronucleus and micronuclei (arrowheads) after fluorescence staining by Hoechst 33342 and acridine orange. **L** A simplified diagram of conjugation process in ciliates. *CV* contractile vacuole; *Ma* macronucleus. Scale bars = 100 μm

Ciliates constitute a morphologically and ecologically diverse lineage (Chi et al. 2021; Duan et al. 2021; Lynn 2008; Ma et al. 2021) that contains both the germline micronucleus (MIC) and somatic macronucleus (MAC) within the single cell (Sheng et al. 2020; Xu et al. 2021; Zhao et al. 2021; Zheng et al. 2021). The MIC is diploid and transcriptionally silent during the vegetative stage, while the MAC is generally polyploid and transcriptionally active, determining the cell’s phenotype (Butler et al. 1984; Prescott 1994; Swart et al. 2013). Ciliates usually reproduce asexually by binary fission when cells are in good environments, while they enter the sexual process under stressed conditions (e.g., starvation). During asexual reproduction, the MAC divides amitotically and the MIC divides mitotically (Katz 2001). In the sexual process of conjugation (Fig. 1L), the MIC undergoes meiosis to form migratory and stationary (gametic) pronuclei, and then, the migratory pronucleus exchanges and fuses with the mating partner’s stationary pronucleus to form the synkaryon. The new MIC and MAC are differentiated from the mitotic products of the synkaryon, while the parental MAC is gradually fragmented and degraded (Raikov 1972, 1982). During the development of the new MAC, large-scale genome rearrangements occur, including DNA elimination, chromosome fragmentation, telomere addition, gene amplification, etc. (Angeleska et al. 2007; Chen et al. 2019; Li et al. 2021; Nowacki et al. 2008; Prescott 1994).

In addition to the ‘regular’ conjugation, some abnormal forms of conjugation occur. The abnormal conjugation between cells with defective MICs (or amicronucleate cells) and cells with functional diploid MICs is called genomic exclusion. This type of abnormal conjugation was first observed in *Tetrahymena thermophila* (Allen 1967) and then reported in *Euplotes raikovi* (Gong et al. 2020). In these two species, the nuclear events during conjugation are very different. In any case, the amicronucleate cells, which are responsible for the abnormal cytogenetic events, represent a valuable genetic tool for some genetic applications, e.g., construction of homozygous strains (Allen 1967).

Difficulties in inducing or observing conjugation have limited the study of sexual processes to a restricted number of ciliate species. As important model ciliates, species of *Paramecium* are among the most studied, especially to assess mechanisms of the sexual process and mating-type determination (Orias et al. 2017; Phadke and Zufall 2009; Singh et al. 2014). However, conjugation has been studied for only one-third of the *Paramecium* species. Furthermore, many of the investigations date back to the middle of the last century and were carried out with equipment and methods that have now been superseded by modern approaches (Fokin et al. 2001; Jankowski 1972). Here, we use such modern approaches to examine a common species of *Paramecium*, and in doing so, we provide new insights into the sexual processes of ciliates.

*Paramecium multimicronucleatum* Powers and Mitchel, 1910 is one of the earliest isolates of this genus. The presence of mating types in *P*. *multimicronucleatum* was reported by Giese (1957), and a circadian rhythm of mating-type reversals was then revealed in some strains (Barnett 1966; Giese 1957), attracting the attention of many researchers. The cytology of conjugation in *P*. *multimicronucleatum* were described by Landis (1925) and Barnett (1964), but the description of conjugation by the two researchers differs. To rectify this, here, we report a detailed description of the nuclear events and their timing during conjugation. We also, for the first time, describe genomic exclusion in *P*. *multimicronucleatum*, which occurs between amicronucleate cells (recently identified and stabilized in our laboratory) and normal micronucleate cells. These results clarify the process of conjugation in *P*. *multimicronucleatum* and provide important data for future in-depth studies of the various mating systems in ciliates.

## Results

### Initiation of conjugation and prezygotic divisions

The two *P*. *multimicronucleatum* strains (dFura23 and dFura24), which were of complementary mating type, were used to study conjugation (Fig. 1). Once starved cells (see “Materials and methods” section) were mixed, they immediately began to form mating pairs (Fig. 1E, K), with a conjugation rate around 85%. Mating pairs gather in the bottom of petri dish forming visible clumps, suitable for obtaining samples of abundant synchronous conjugants. This initial mixing time was taken as time 0 of the process.

**Fig. 2:**
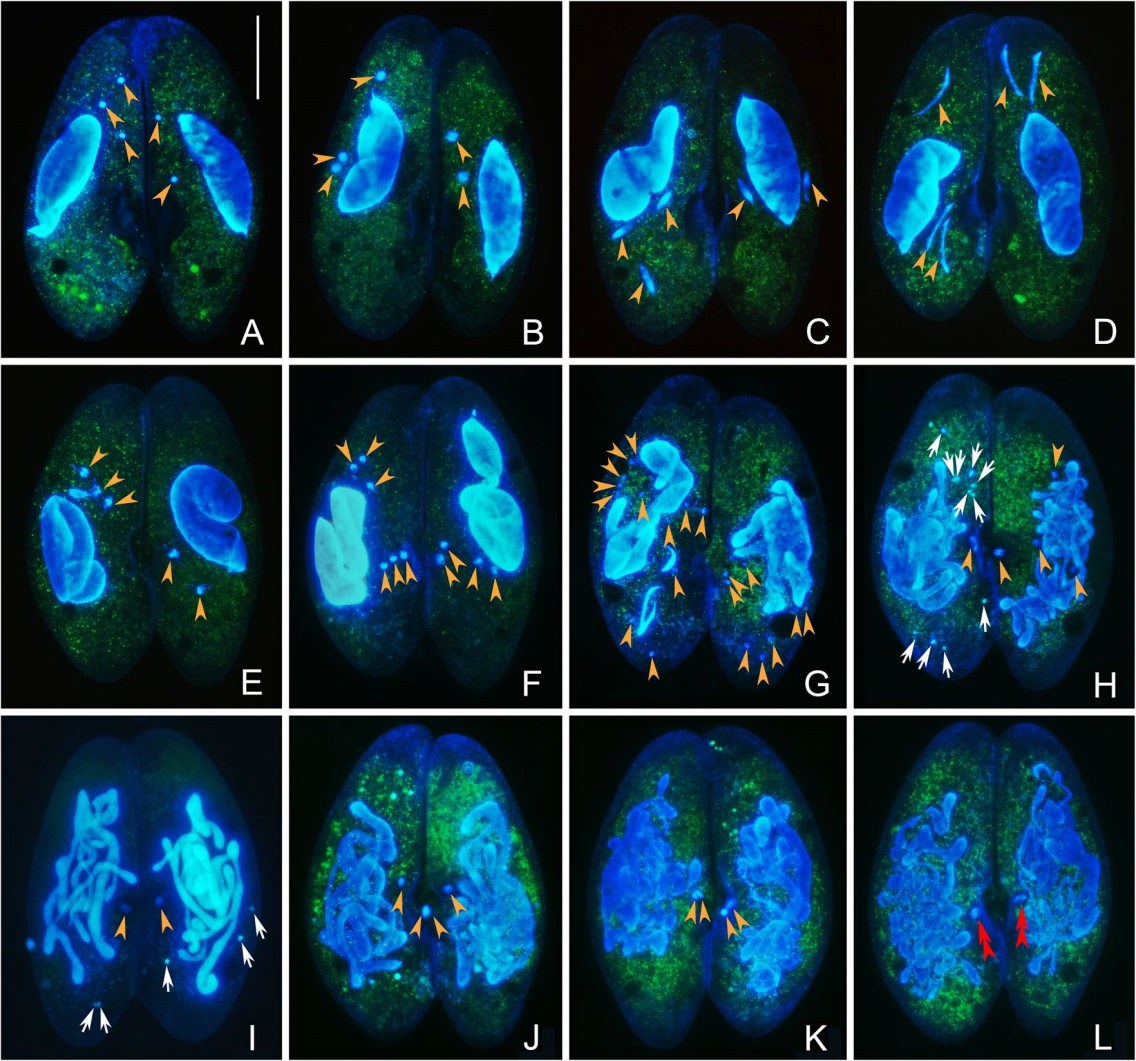
Prezygotic divisions and synkaryon formation in *Paramecium multimicronucleatum* after fluorescence staining by Hoechst 33342 and acridine orange. **A** In conjugation pairs formed just after mixing (time 0), the morphology of MAC and MICs does not change. **B** MICs are enlarged. **C** MICs elongate into fusiform shape. **D** The “crescent” stage in prophase of meiosis I. **E** Metaphase of meiosis I: chromosomes are arranged neatly on the equatorial plate. The degeneration of MAC starts to be observed. **F** The end of anaphase I of meiosis I: chromosomes have been gathered to cell bipolar to a large extent. **G** Telophase of meiosis II, after which 12/8 nuclear products are produced. **H, I** Variable number of MICs undergoes the third prezygotic division (mitosis) and the rest degenerates. **J** Nuclear products of the third prezygotic division near the paroral cone remain as migratory and stationary pronuclei. **K** The exchange of migratory pronuclei is completed, and they are going to fuse with the stationary one. **L** The migratory and stationary pronuclei fuse to form synkaryon and prepare for the first postzygotic division. Orange arrowhead: MIC and its products of prezygotic divisions before the formation of synkaryon. Red double arrowheads: synkaryon. White arrow: degenerating nucleus. Scale bar = 80 μm

The two cells in the mating pair typically have either two or three micronuclei (MICs), so we use this nuclear pattern (one cell with two MICs and one with three MICs) to describe the process. At the beginning of conjugation, the morphology of both the macronucleus (MAC) and the MICs remain unchanged (Fig. 2A). Then, the MICs enlarge and elongate gradually, to enter the "crescent stage" of the first prezygotic division (meiosis I) (Fig. 2B–D). This initial step is about 5.5 h, followed by the "crescent stage" of the MICs which lasts about 1.5 h. Subsequently, the MICs enter the metaphase of meiosis I, during which the entire nucleus is typically fusiform and MIC chromosomes are arranged on the equatorial plate (Fig. 2E). After another 2.5 h, the MICs conclude the first meiotic division, resulting in four or six nuclear products (Fig. 2F).

The four–six haploid nuclei then enter the second meiotic division (the second prezygotic division), which lasts about 0.5 h with the production of eight or 12 nuclei (Fig. 2G). Variable numbers of these nuclei (we observed up to six nuclei) presumably possess the potential to produce functional pronuclei. At least one of these nuclei divides again by mitosis (third prezygotic division), which takes about 0.5 h. In the next 0.5 h, the mitotic products near paroral cone remain as the migratory and stationary pronuclei, while the others become pyknotic and eventually disappear (Fig. 2H, I).

### Synkaryon formation

Fertilization occurred when the migratory pronucleus migrates from one cell to the other and fuses with the stationary pronucleus to form the synkaryon (Fig. 2J–L). This step, from the formation of gametic pronuclei to their fusion in the synkaryon, takes about 0.5 h.

### Postzygotic divisions

**Fig. 3:**
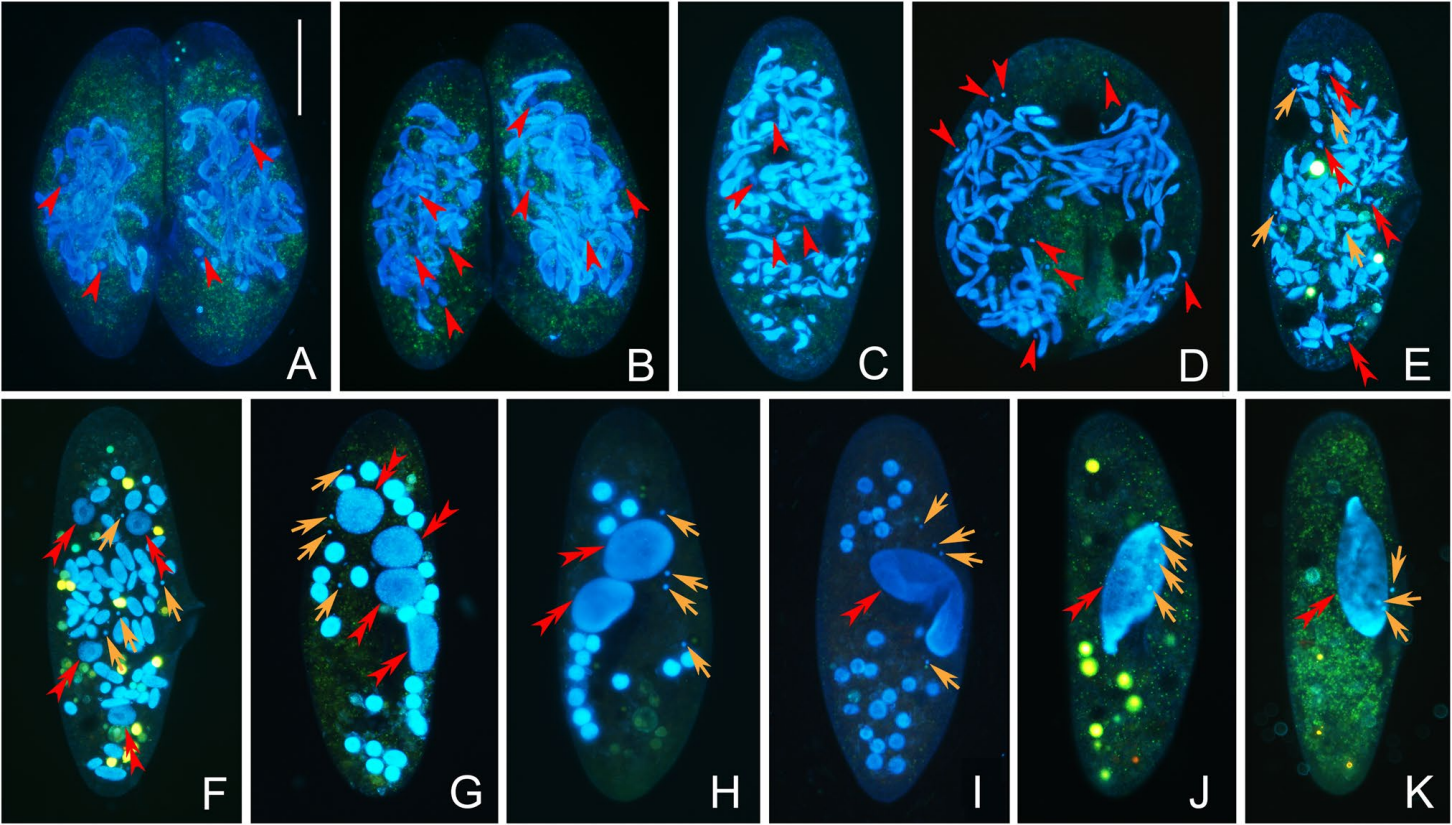
Nuclear events of postzygotic and cell divisions in *Paramecium multimicronucleatum* after fluorescence staining by Hoechst 33342 and acridine orange. **A** The first postzygotic division ends, and there are two products in each conjugant. **B** The second postzygotic division ends, and there are four products in each conjugant. **C** Four products of the second postzygotic division are entering to the third postzygotic division and conjugant pair separates after the second postzygotic division. **D** The third postzygotic division ends, and there are eight products in each conjugant. The cell inflates to be round shape for a while. **E**–**G** Eight nuclear products in each exconjugant differentiate into four MAC anlagen and four MIC anlagen. **H** After the first cell division, there are two MAC anlagen and four MIC anlagen in each exconjugant. **I** After the second cell division, there are one MAC anlagen and four visible MIC anlagen in each exconjugant. Some fragments of parental MAC remain at this stage. **J, K** Cells have been recovered into the vegetative stage. Red arrowhead: nuclear products of synkaryon. Red double arrowheads: MAC or MAC anlagen. Orange arrow: MIC or MIC anlagen. Scale bar = 80 μm

After fertilization, the synkaryon divides mitotically twice to form four nuclear products in each cell (Fig. 3A, B). The two conjugants separate after the second postzygotic division (Fig. 3C). In each exconjugant cell, the four nuclear products divide again to form eight nuclei. During this division step, the cell swells and assumes a round and larger shape for about 0.5 h (Fig. 3D). Subsequently, the eight nuclei in each cell differentiate into four MAC anlagen and four MIC anlagen (Fig. 3E). The MAC anlagen swell gradually during the development, and early on they are recognizable as they contain a poorly stained central area and numerous deep stained dots (Fig. 3F, G).

When the development of the MAC anlagen is well under way, about 36 h after mixing (Fig. 3G), refeeding is necessary for conjugation to continue. After about 84 h after instigating conjugation, the cell undergoes the first binary fission. While the four MAC anlagen distribute equally into the two daughter cells, the four MICs divide mitotically, so that each daughter cell receives four products of MICs’ division (Fig. 3H). The fragments of parental MAC (distinguished from MAC anlagen by their darker color) are randomly and passively distributed into the two daughter cells. Subsequently, the four MICs divide concomitantly with the second binary fission. At the end, each daughter cell contains one MAC anlagen and four MIC (Fig. 3I, J), and it then looks like a typical vegetative cell, although some fragments of the parental MAC are still present.

When cells divide by binary fission, the new MAC and MICs undergo amitosis and mitosis, respectively, while the remaining fragments of parental MAC gradually disappear. After several rounds of binary fission, some cells may lose copies of the MICs, resulting in various number of MICs in different individuals (Fig. 3K).

### Fate of the parental MAC

The parental MAC starts to disintegrate following metaphase of the first meiotic division (Fig. 2E). At the end of the third prezygotic division, the shape of the parental MAC changes from a leaf form into a tangle of cords (Fig. 2H, I). After the second postzygotic division, when the two cell partners separate, the parental MAC starts to break into many rounded or slightly prolonged fragments of diverse dimensions (Fig. 3C). With the development of the MAC anlagen, these fragments become irregular and finally disappear.

### Cytogenetics of genomic exclusion

**Fig. 4:**
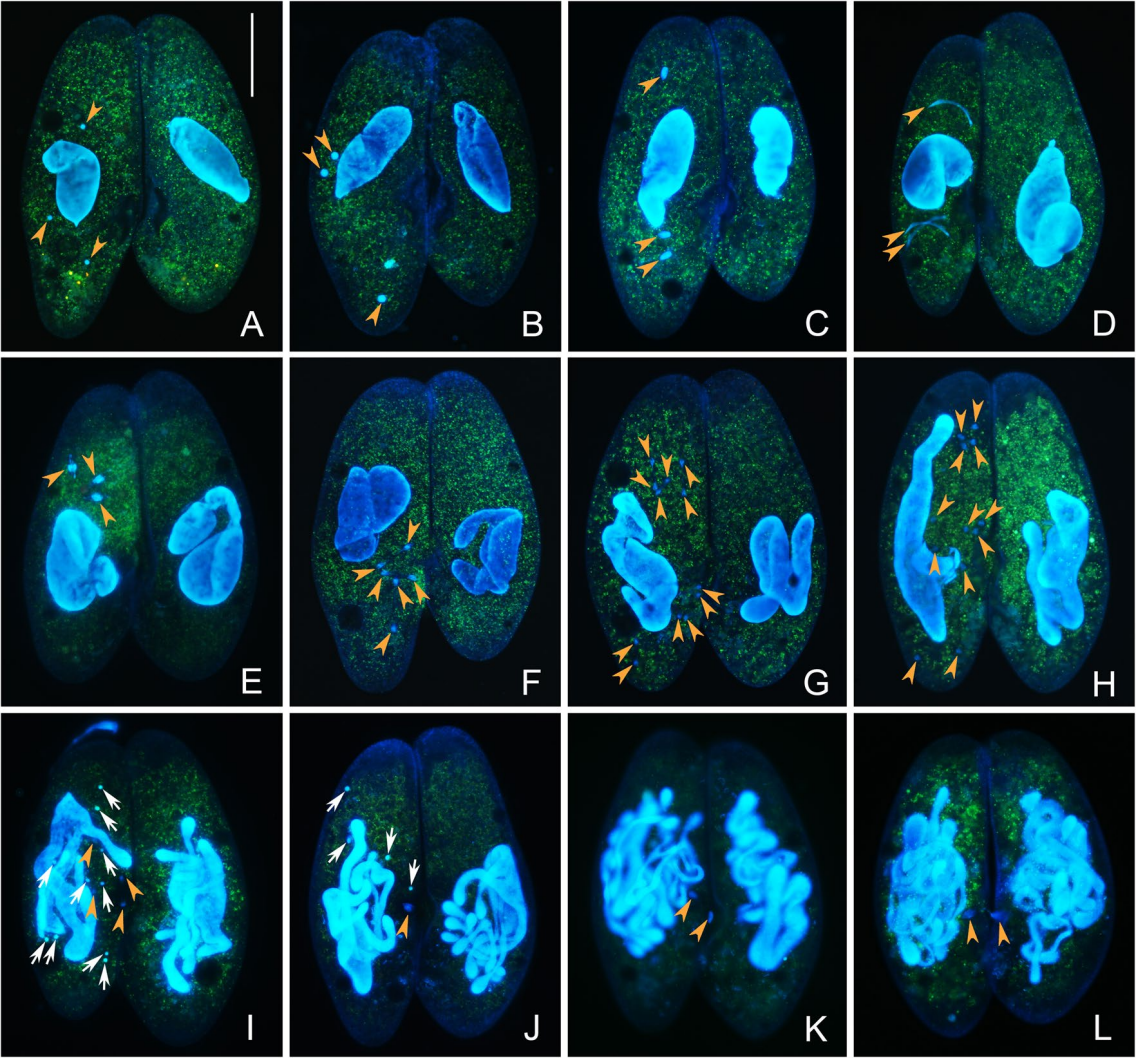
Nuclear events before postzygotic divisions of genomic exclusion occurring between amicronucleate and micronucleate cells in *Paramecium multimicronucleatum* (after fluorescence staining by Hoechst 33342 and acridine orange). **A**–**K** Three prezygotic divisions (miosis I, miosis II, and mitosis) only occur in the micronucleate cell, as in normal conjugation shown in Fig. 2. **L** The micronucleate conjugant gives rise to migratory and stationary haploid nuclei, and the migratory one migrates to the amicronucleate conjugant. Orange arrowhead: MIC and its products of prezygotic divisions before the formation of new diploid nucleus. White arrow: degenerating nuclei. Scale bar = 80 μm

When amicronucleate and normal cells conjugated, the MICs in the normal cell (dFura24) perform the three prezygotic divisions leading to the production of migratory and stationary pronuclei (Fig. 4). Then, the migratory pronucleus moves from the normal micronucleate conjugant to the amicronucleate one, and no fertilization occurs; the result of this is that each exconjugant contains a haploid gametic nucleus (Fig. 4L). We speculate that micronuclear diploidy is then restored following transfer of the haploid gametic nucleus, but we have not been able to observe the details of this: possibly diploidy is restored in both conjugants by endoreplication; alternatively, the haploid nucleus undergoes another round of mitosis and then fuses to restore diploidy. Regardless, of the details, the separation of the conjugants and the process of postzygotic divisions are the same as the normal conjugation, as shown in Fig. 3.

## Discussion

### Nuclear events during conjugation in *Paramecium multimicronucleatum*

**Fig. 5:**
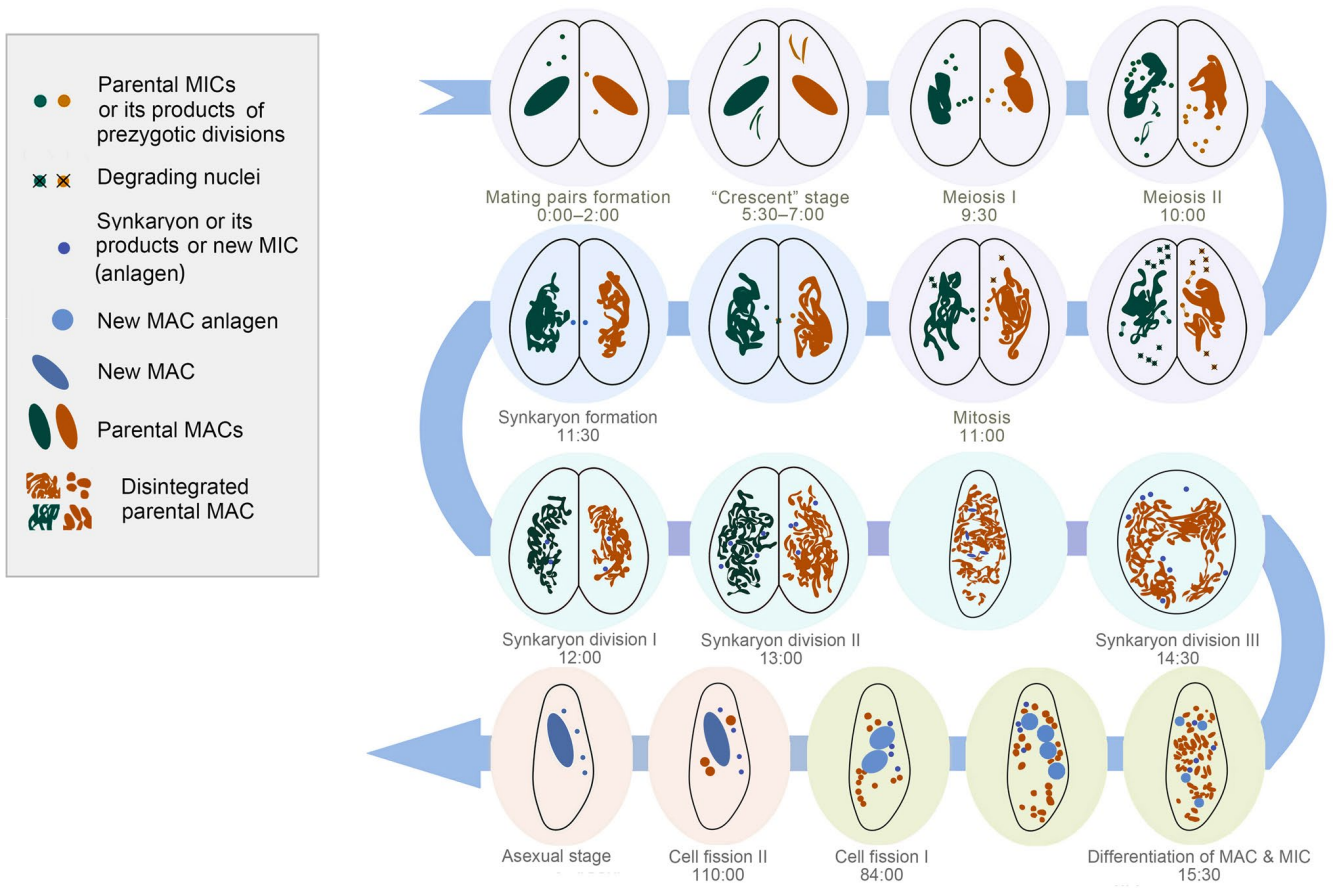
Morphology and timing of nuclear events during conjugation in *Paramecium multimicronucleatum*

Our results provide evidence that the nuclear events occurring during conjugation between *P*. *multimicronucleatum* cells of strains dFura23 and dFura24 include three prezygotic micronuclear (MIC) divisions (meiosis I, meiosis II, and mitosis), three postzygotic synkaryon divisions, and two successive cell fissions. The whole process takes about 110 h (Fig. 5).

A description of the nuclear events in *P*. *multimicronucleatum* conjugation was first reported by Landis (1925). According to Landis, the sexual process includes three prezygotic MIC divisions (meiosis I, meiosis II, and mitosis), six postzygotic nuclear divisions, and two successive cell fissions. Our observations support only in part the previously described process. The main differences include: (1) the number of nuclei that presumably possess the potential to produce functional pronuclei is four according to Landis (1925), while we found that it was variable (one to six); (2) our observations of the postzygotic nuclear divisions differed from those previously reported. Landis (1925) reported seven of the eight synkaryon products of the third postzygotic division degrade, and the surviving one divides twice more, resulting in four products that differentiate into two MIC and two macronuclear (MAC) anlagen; then, each MIC and MAC anlage divides again before the first cell division. Conversely, our observations show that the eight synkaryon products of the third postzygotic divisions differentiate directly into four MIC and four MAC anlagen before the first cell division. There are at least two arguments supporting our finding. First, no degeneration of zygotic nuclear products was observed in samples collected every half hour from the third postzygotic division. For this analysis, the combined acridine orange (AO) and Hoechst 33342 (HO) staining was used, as it allows recognition of the degraded (turquoise) and the non-degraded (blue) nuclei (Fig. 2H, I and Fig. 4I, J). Second, the stage with “two MIC and two MAC anlagen” was never observed in the present work. After the third postzygotic division, there is only about 1 h before the invisible differentiation of MIC and MAC anlagen, and no division of these anlagen was observed in our work. Conversely, all exconjugants have four MIC and four MAC anlagen (Fig. 3D, E), which clearly come from the initial differentiation of the nuclear products of the third postzygotic division.

**Fig. 6:**
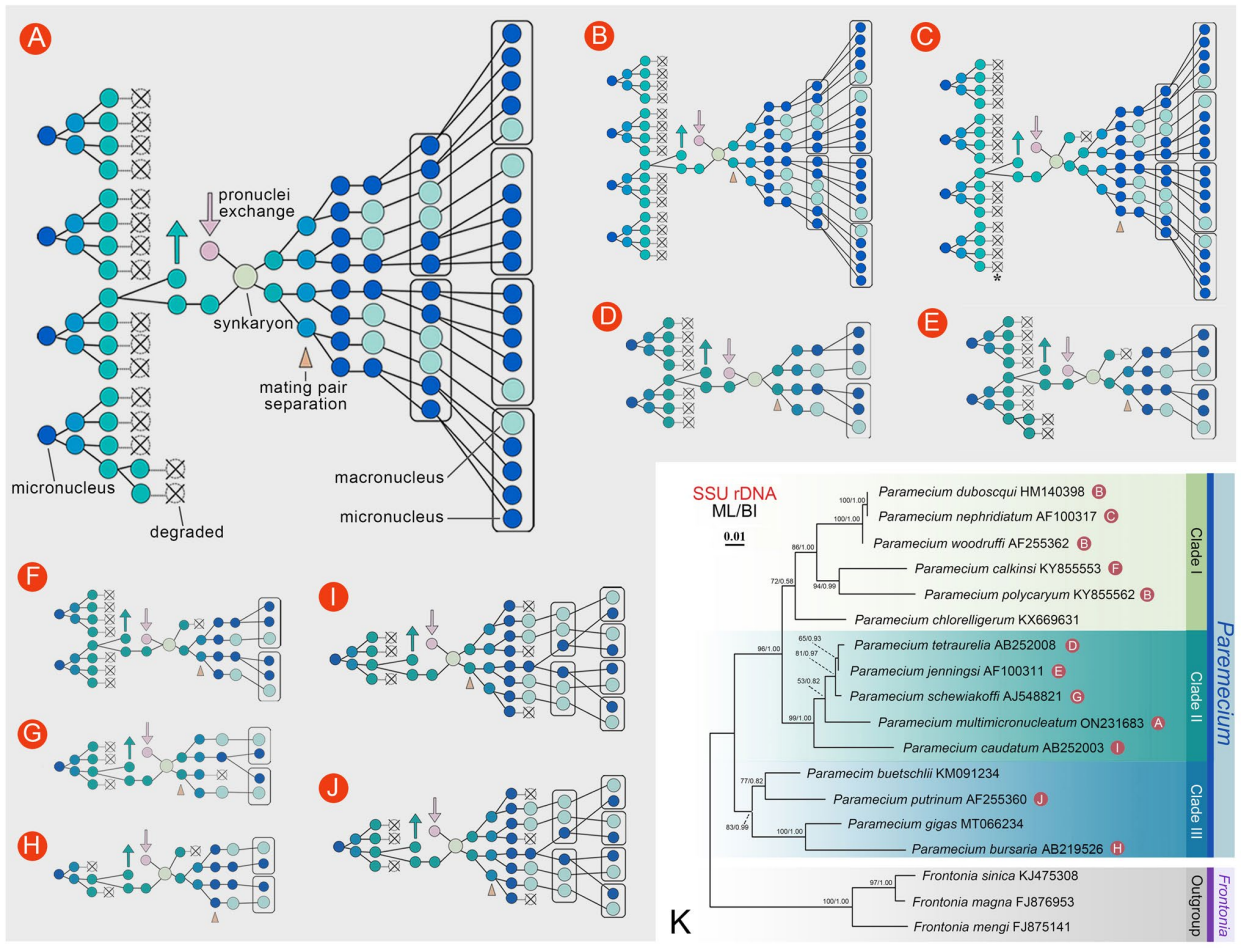
Different patterns of nuclear events during conjugation in *Paramecium* species and phylogenetic tree based on SSU rRNA gene sequences. **A** *P*. *multimicronucleatum*, from the present study; **B** *P*. *duboscqui* (Watanabe et al. 1996), *P*. *polycaryum* (Diller 1958), and *P*. *woodruffi* (Fokin et al. 2001); **C** *P*. *nephridiatum* (Jankowski 1961); **D** *P*. *aurelia* complex (Diller 1936; Fokin et al. 2001; Sonneborn 1938); **E** *P*. *jenningsi* (original data, unpublished); **F** *P*. *calkinsi* (Nakata 1956); **G** *P*. *schewiakoffi* (Fokin et al. 2001); **H** *P*. *bursaria* (Wichterman 1948); **I** *P*. *caudatum* (Yang et al. 2007); **J** *P*. *putrinum* (Jankowski 1972). **K** Maximum-likelihood (ML) tree of the genus *Paramecium* based on SSU rRNA gene sequences. *Frontonia magna* (FJ876953), *F*. *mengi* (FJ875141), and *F*. *sinica* (KJ475308) were used as outgroup species. Numbers at nodes represent the bootstrap values of ML out of 1000 replicates and posterior probability of Bayesian analysis (BI). The scale bar corresponds to one substitution per 100 nucleotide positions. Triangle: separation of the conjugants. Arrow: exchange of migratory pronuclei. Box: daughter cell after cell division. Asterisk: more than two pronuclei could appear (Fokin et al. 2001; Jankowski 1961)

The cytology of conjugation in *P*. *multimicronucleatum* was also reported by Barnett (1964), who described that only the nucleus in the paroral cone undergoes the third prezygotic division. Although only one nucleus divides at this stage in most *Paramecium* species whose conjugation have been described (Fokin et al. 2001; with the exception of *P*. *jenningsi* (original data, unpublished) and *P*. *nephridiatum* (Jankowski 1961)) (Fig. 6), our study indicates that in *P*. *multimicronucleatum*, this number is not always one. These different observations could be attributed to errors due to the limitations of experimental conditions, such as the presence of food vacuoles or maternal MAC fragments which can obscure some nuclear products (Barnett 1964). Other explanations for the difference could be attributed to some variation in the conjugation process among different strains, or they are in fact different species due to the wrong species identification.

### Comparison of the nuclear events during conjugation in different *Paramecium* species

MAC and MIC development during conjugation in *Paramecium* has been studied for over 100 years (Hamburger 1904; Jankowski 1972; Watanabe et al. 1996). By comparing the conjugation process in different *Paramecium* species, three key points emerge; each of these is outlined below.

First, despite that the number of MIC varies among species, there are always three prezygotic divisions. All the MICs undergo the first and the second prezygotic divisions, while only one selected nucleus (or several nuclei in *P*. *multimicronucleatum* and *P*. *jenningsi*) undergoes the third prezygotic division. The only exception to this scheme is represented by *P*. *bursaria* (Fig. 6H), as only one nucleus is selected at the end of each division to complete the process (Wichterman 1948).

Second, there are some diversities among species in the patterns of postzygotic nuclear divisions and the following cell divisions. Our observations suggest that the diversities in the number of postzygotic and cell divisions, as well as the number of offspring individuals, depend mainly on the number of MICs in vegetative cells and nuclear products degraded after each division. Before MIC and MAC differentiation, there are generally three rounds of synkaryon divisions, exceptionally four in *P*. *nephridiatum* (Fig. 6C) (Jankowski 1961), and two in *P*. *tetraurelia* (Fig. 6D) (Diller 1936; Sonneborn 1938) and *P*. *schewiakoffi* (Fig. 6G) (Fokin et al. 2001)*.* The number of degenerating nuclei after each synkaryon division also varies among species. For *P*. *nephridiatum* (Fig. 6C) (Jankowski 1961), *P*. *jenningsi* (Fig. 6E) (original data, unpublished), *P*. *calkinsi* (Fig. 6F) (Nakata 1956), and *P*. *bursaria* (Fig. 6H) (Wichterman 1948), one product of the first postzygotic division degrades. One product of the second synkaryon division degenerates in *P*. *schewiakoffi* (Fig. 6G) (Fokin et al. 2001), while three of the eight products after the third synkaryon division degenerate in *P*. *caudatum* (Fig. 6I) (Yang et al. 2007) and *P*. *putrinum* (Fig. 6J) (Jankowski 1972). For other species, no degeneration occurs for the postzygotic division products. As a result of postzygotic divisions and degeneration, cells have to divide one or two times to recover to the vegetative stage.

Third, exconjugant cells separate at three different time points, for example: (i) after the first postzygotic division in *P*. *tetraurelia* (Diller 1936; Fokin et al. 2001; Sonneborn 1938), *P*. *woodruffi* (Jankowski 1961), and *P*. *duboscqui* (Watanabe et al. 1996); (ii) after the second postzygotic division in *P*. *multimicronucleatum*; and (iii) after the fourth postzygotic division in *P*. *nephridiatum* (Fokin et al. 2001; Jankowski 1961).

The identified variations in the conjugation process among different *Paramecium* species are not directly related to evolution within the genus, as shown by mapping these variations into the phylogenetic tree based on SSU rRNA gene sequence (Fig. 6). For instance, species with the same pattern of mating pair separation do not cluster together, indicating that time points of mating pair separation may be a species-specific character. According to the present and previous analysis (Fokin et al. 2001), we conclude that the different patterns of nuclear events in *Paramecium* have evolved independently and do not reflect phylogenetic relationships, a phenomenon known as mosaic evolution or evolutionary heterochrony (Corliss 1975; Fokin et al. 2001; Jankowski 1972; Raikov 1982).

### Genomic exclusion in ciliates

Genomic exclusion was originally described in *Tetrahymena thermophila* (Allen 1967). In this species, genomic exclusion comprises two rounds of conjugation. In the first round, the mating pairs separate prematurely, following unidirectional exchange of a gametic pronucleus from the micronucleate cell to the defective partner, giving rise to the exconjugants with homozygous MIC and their original MAC (heterokaryons). The exconjugants are therefore sexually mature and can enter into the second round of conjugation, which proceeds normally and gives rise to cells that are whole-genome homozygotes. An abnormal conjugation between amicronucleate and micronucleate cells was also reported in *Euplotes raikovi* (Gong et al. 2020). However, in *E*. *raikovi*, the micronucleate partner does not exchange the gametic pronucleus with the defective one. Instead, the migratory pronucleus fuses with the stationary one in the same cell to generate the synkaryon, and after conjugation, the amicronucleate cell remains without MIC.

Genomic exclusion thus represents an ideal method for obtaining homozygotes which is essential for studying the function of alleles, much easier and faster than the traditional ways. However, amicronucleate or MIC defective cells are essential for this process. Amicronucleate cells can be collected from the wild or have been obtained from *Oxytricha hymenostoma*, *P*. *caudatum*, *P*. *tetraurelia*, *P*. *jenningsi*, *T*. *thermophila*, *E*. *rakovi*, *Pseudourostyla levis*, and *Stylonychia lemnae* using micropipetting, nitrosoguanidine mutagenesis, or other methods (Allen 1967; Ammermann et al. 1989; Dawson 1919; Gong et al. 2020; Landis 1920; Mikami 1979; Ng SF 1989; Takahashi and Suhama 1991). We are confident that the new and stable amicronucleate cell line established from the wild-type strain dFura23 of *P*. *multimicronucleatum* syngen 2 will find applications to study the biology of *Paramecium*.

## Materials and methods

### Cell culture, conjugation induction, and cell staining

Mating-type complementary strains dFura23 and dFura24 of *Paramecium multimicronucleatum* syngen 2 were kindly supplied by Professor Masahiro FUJISHIMA at Yamaguchi University (http://nbrpcms.nig.ac.jp/paramecium/strain/?lang=en). A stable amicronucleate strain of *P*. *multimicronucleatum* syngen 2 spontaneously arose in our monoclonal cultures of the wild-type strain dFura23 under normal culture conditions and was then isolated.

The ciliates were cultured in 5% fresh lettuce juice that was diluted with modified Dryl solution using KH_2_PO_4_ instead of NaH_2_PO_4_ (KDS) (Dryl 1959; Yang et al. 2007), inoculated with the bacterium *Klebsiella pneumoniae*. Ciliates used in the analysis are in a state of mild starvation; their conjugative activity lasts for about 48 h after the culture medium becomes clear (i.e., with very few bacteria).

Cells of different mating types start to form mating pairs within a minute after mixing, so the mixing time was considered as the time 0. Cell samples were then collected every 30 min or 1 h and then stained with Hoechst 33342 (HO) (Beyotime Institute of Biotechnology, Jiangsu, China) and acridine orange (AO) (Shanghai Chemical Reagent Co., Ltd., Shanghai, China) (Yang et al. 2007). For every 100 μl of cell suspensions, 1.8 μl Hoechst 33342 (HO, 2 mg/ml) and 0.8 μl acridine orange (AO, 100 μg/ml) were used. The mixtures were incubated at 25 ± 1 °C for 20 min, and cells were observed under a “ZEISS AXIO Imager D2” fluorescence microscope, equipped with an Axiocam 506 camera for photographic documentation. For each time point, 30–50 mating pairs were recorded (Gong et al. 2020; Jiang et al. 2019).

### DNA extraction, PCR amplification, and sequencing

Genomic DNA was extracted using MagAttract^®^ HMW DNA Kit (QIAGEN, Germany, Cat. No.: 67563) following the manuals. The sequence of the small subunit ribosomal RNA (SSU rRNA) gene was obtained by amplification with universal primers 18SF (5′-AACCTGGTTG ATCCTGCCAGT-3′) and 18SR (5′-TGATCCTTCTGCAGGTTCACCTAC-3′) (Jerome et al. 1996; Medlin et al. 1988) using Q5^®^ Hot Start High-Fidelity DNA Polymerase (New England BioLabs, USA). The PCR products were sequenced bidirectionally by Tsingke Biological Technology Company (Qingdao, China).

### Phylogenetic analyses

Phylogenetic analyses were performed using SSU rRNA gene of *P*. *multimicronucleatum* strain dFura23 and other 17 SSU rRNA gene sequences which were obtained from GenBank (accession number as shown in Fig. 6). Sequences were aligned using the GUIDANCE2 Server (http://guidance.tau.ac.il/) with default parameters (Sela et al. 2015). The alignment was manually modified using BioEdit v.7.0.1 (Hall 1999), resulting in a matrix of 18 taxa with 1660 nucleotide sites. Both maximum-likelihood (ML) and Bayesian inference (BI) analyses were performed in CIPRES Science Gateway (http://www.phylo.org/sub_sections/portal). The ML tree was constructed using RAxML-HPC2 on XSEDE v.8.2.12 with the GTRGAMMA model and 1000 bootstrap replicates, while the BI analysis was performed using MrBayes on XSEDE v.3.2.6 with the GTR + I + G model which was selected by MrModeltest v.2.0 (Nylander 2004; Stamatakis 2014). Markov chain Monte Carlo (MCMC) simulations were run for 10^6^ generations with a frequency of 100 generations and a burn-in of 10^4^ trees. Tree topologies were visualized with MEGA v.7.0.26 (Kumar et al. 2016).

## Data Availability

The small subunit ribosomal RNA gene sequence generated during the present study has been deposited to GenBank with accession number: ON231683 (https://www.ncbi.nlm.nih.gov/nuccore/ON231683). The other data generated during this study are included in this published article.
